# Effect of a Feedback Visit and a Clinical Decision Support System Based on Antibiotic Prescription Audit in Primary Care: Multiarm Cluster-Randomized Controlled Trial

**DOI:** 10.2196/60535

**Published:** 2024-12-18

**Authors:** Pauline Jeanmougin, Stéphanie Larramendy, Jean-Pascal Fournier, Aurélie Gaultier, Cédric Rat

**Affiliations:** 1 Department of General Practice Faculty of Medicine Nantes University Nantes France; 2 Antibioclic Steering Committee Paris France; 3 POPS - SFR ICAT University of Angers Angers France; 4 Methodology and Biostatistics Platform Nantes University Hospital Nantes France

**Keywords:** antibacterial agents, feedback, clinical decision support system, prescriptions, primary health care, clinical decision, antibiotic prescription, antimicrobial, antibiotic stewardship, interventions, health insurance, systematic antibiotic prescriptions

## Abstract

**Background:**

While numerous antimicrobial stewardship programs aim to decrease inappropriate antibiotic prescriptions, evidence of their positive impact is needed to optimize future interventions.

**Objective:**

This study aimed to evaluate 2 multifaceted antibiotic stewardship interventions for inappropriate systemic antibiotic prescription in primary care.

**Methods:**

An open-label, cluster-randomized controlled trial of 2501 general practitioners (GPs) working in western France was conducted from July 2019 to January 2021. Two interventions were studied: the standard intervention, consisting of a visit by a health insurance representative who gave prescription feedback and provided a leaflet for treating cystitis and tonsillitis; and a clinical decision support system (CDSS)–based intervention, consisting of a visit with prescription feedback and a CDSS demonstration on antibiotic prescribing. The control group received no intervention. Data on systemic antibiotic dispensing was obtained from the National Health Insurance System (*Système National d’Information Inter-Régimes de l’Assurance Maladie*) database. The overall antibiotic volume dispensed per GP at 12 months was compared between arms using a 2-level hierarchical analysis of covariance adjusted for annual antibiotic prescription volume at baseline.

**Results:**

Overall, 2501 GPs were randomized (n=1099, 43.9% women). At 12 months, the mean volume of systemic antibiotics per GP decreased by 219.2 (SD 61.4; 95% CI −339.5 to −98.8; *P*<.001) defined daily doses in the CDSS-based visit group compared with the control group. The decrease in the mean volume of systemic antibiotics dispensed per GP was not significantly different between the standard visit group and the control group (−109.7, SD 62.4; 95% CI −232.0 to 12.5 defined daily doses; *P*=.08).

**Conclusions:**

A visit by a health insurance representative combining feedback and a CDSS demonstration resulted in a 4.4% (-219.2/4930) reduction in the total volume of systemic antibiotic prescriptions in 12 months.

**Trial Registration:**

ClinicalTrials.gov NCT04028830; https://clinicaltrials.gov/study/NCT04028830

## Introduction

Antibiotic resistance is a worldwide threat to public health and has major consequences for human health and health care systems [1,2]. Recently, the literature has advocated antimicrobial stewardship programs to reduce inappropriate antibiotic prescriptions and improve adherence to medical guidelines in primary care [3]. Several levers have been proposed to change behavior: active or passive training [4], audits and clinical practice feedback [5], patient-focused actions, nudge interventions [6], and financial incentives [7]. Another effective intervention combines an audit of clinical practice and promoting appropriate and necessary antibiotic prescription [8-10]. Although the literature advocates for multifaceted interventions, results vary depending on how these interventions are implemented [7,8,11-13].

Obstacles to implementing relevant interventions include reaching health care professionals and identifying the specific barriers and facilitators that drive behavioral change [14,15]. In France, this task is the responsibility of health insurance representatives (HIRs) who work for the National Health Care Insurance Organization (NHIS) known as Assurance Maladie. This organization provides health care insurance to approximately 90% of French citizens of all ages. HIRs visit general practitioners (GPs) 3 or 4 times a year to promote actions related to public health priorities. However, evidence is lacking on whether these visits do, in practice, have a positive effect on prescribing habits among primary care providers.

In this context, recent studies suggest that clinical decision support systems (CDSS) can help physicians make appropriate decisions when prescribing antibiotics [16,17]. However, their effectiveness in reducing the volume of antibiotics prescribed, and whether they improve the overall quality of prescriptions, has rarely been evaluated at the population scale. Among the several CDSSs available [18-20] to optimize antibiotic prescribing, French GPs most often use Antibioclic [21-23]. The system was developed by French academics and released in 2011, and it provides easy access to best practice recommendations.

This study aimed to evaluate the effect of a multifaceted intervention involving an HIR visit, clinical practice feedback, and the presentation of an online decision aid (the CDSS-based visit group) on antibiotic prescribing among GPs in western France. The intervention was compared with (1) a standard intervention involving a HIR visit, clinical practice feedback, and a prescribing information leaflet (the standard visit group) and (2) no intervention (the control group).

## Methods

### Study Design

The study was an open-label, cluster-randomized controlled trial. It was conducted between July 2019 and January 2021 among GPs in western France (the Pays de la Loire geographic area, with 3,832,120 inhabitants). The academic research team worked in collaboration with HIRs working for the regional division of the NHIS.

This study followed the CONSORT (Consolidated Standards of Reporting Trials) reporting guidelines (Multimedia Appendix 1) and was registered with ClinicalTrials.gov under NCT04028830 (version 5, April 19, 2024).

### Participants and Setting

All 2758 GPs working in private practice in the Pays de la Loire geographical area were considered eligible if they had been practicing in April 2019 and had seen at least 100 different patients in 2018. GPs were excluded if they practiced alternative medicine, including acupuncture, allergology, or angiology; they were participating in another national antibiotic resistance project; or they were involved in developing the intervention.

### Interventions

In total, 2 multifaceted interventions were evaluated in this study. The group of HIRs were collectively trained on June 17, 2019, before the interventions.

In the CDSS-based visit group, the intervention was carried out by the regional HIR at the GPs’ practice and consisted of (1) providing information about antibiotic resistance, good antibiotic use, and prescription practices; (2) giving feedback based on individual, regional, and national antibiotic prescription rates; and (3) providing a presentation on how to use the CDSS in the treatment of cystitis and tonsillitis. The CDSS is presented in Multimedia Appendices 2 and 3 The user selects the pathology (not limited to tonsilitis or urinary tract infections [21]), and the tool suggests a therapeutic strategy adapted to French national recommendations. Access to Antibioclic is free of charge.

The standard intervention (standard visit group) was also carried out by the HIR at the GPs’ practice. It consisted of (1) providing information about antibiotic resistance, good antibiotic use, and prescription practices; (2) giving feedback based on individual, regional, and national antibiotic prescription rates; and (3) providing an information leaflet about the appropriate antibiotic treatment for cystitis and tonsillitis (Multimedia Appendix 4). The control group received a routine visit by the regional HIR, but the discussion focused on a health priority other than antibiotic prescription.

Visits were planned between July 2019 and January 2020.

### Randomization

Once eligible GPs had been included, they were randomly assigned at a 1:1:1 ratio to either the CDSS-based visit group, the standard visit group, or the control group (in July 2019). GPs were clustered within practices to avoid contamination bias stemming from shared tracking mechanisms and communication among GPs within the same practice.

The regional division of the NHIS was responsible for selecting GPs according to the inclusion criteria, grouping practices according to their location, and assigning GPs to the randomization arm to which their practice had been allocated.

The assessment of outcomes may be considered formally blinded, as all trial-relevant data were collected with automated processes used to record health insurance claims information.

### Outcomes

The primary outcome was the total volume of systemic antibiotics dispensed as defined daily doses (DDD; according to the World Health Organization) per participating GP at the end of 12 months of follow-up. All systemic antibiotics listed in the 2017 Anatomical Therapeutic Chemical classification system were included (Anatomical Therapeutic Chemical class J01) [24]. Patients treated with topical antibiotics, antituberculosis agents, antiparasitic agents, or antimycotic agents were excluded.

Secondary outcomes were the total volume of systemic antibiotics dispensed after 3 months of follow-up; the volume of antibiotics considered critical at the European level according to the Aware classification [25,26] (third-generation cephalosporins, fluoroquinolones, and amoxicillin-clavulanic acid); and the volume of antibiotics dispensed in 2 specific patient subgroups (people aged >65 years and children aged <6 years). In France, these 2 populations are prescribed a high volume of antibiotics [27].

### Data Collection

All data were extracted from the NHIS database (*Système National d’Information Inter-Régimes de l’Assurance Maladie,* SNIIRAM) using routine procedures.

To describe the population included in the study, the following variables characterizing GPs were extracted at baseline: (1) age, sex, practice location, type of practice (group or sole practitioner), and patient base (number of patients on their list at the beginning of the intervention), and (2) the number of consultations and antibiotic dispensing data over the year preceding the start of follow-up.

Antibiotic dispensing data (required to calculate primary and secondary outcome measures) were collected over a 12-month period starting from the date of the HIR visit. The SNIIRAM database holds no clinical data (ie, there is no information about a patient’s diagnosis or clinical indications).

For GPs who did not receive an HIR visit (refusal, unavailability, etc), a follow-up start date was selected at random from among the actual dates of visits made by HIRs to other GPs.

### Statistical Analysis

The minimum number of participants was calculated on the basis of preliminary data. The number of eligible GPs was estimated at 2400. The average volume of antibiotics dispensed per GP was estimated at 7671 (SD 5360) DDD. A previous study [28] that investigated a subdivision of the study region found that the average number of GPs per practice was 1.9. We assumed an intraclass correlation coefficient of 0.05. With an α risk of 5% and a power of 80%, a significant result could be demonstrated if an average difference of 450 DDD per GP was identified between the 2 arms.

The analysis was performed on an intention-to-treat basis among all GPs practicing at the time of randomization (refer to the research protocol, Multimedia Appendix 5). The aim was to conduct a “real-life” study that considered the fluctuating impact of the same intervention received by GPs.

The data collection process minimized missing data. The overall volume of antibiotics dispensed after 12 months of follow-up for GPs who ceased their activity (retirement, moved house, death, etc) during the follow-up period was imputed using a multiple imputation method.

The statistical unit was the GP. GPs and their patients were described using numbers and percentages of each modality for qualitative variables, and by means and SD for quantitative variables, both overall and according to the 3 randomization arms.

For the primary end point, a hierarchical procedure was used to compare the 3 arms, while maintaining a 5% α risk. First, we tested for differences between the CDSS-based visit group and the control group. If this test was significant, a second test was performed to compare the standard visit group with the control group.

Bivariate analyses were used to select variables to be included in the multiple imputation. These concerned the characteristics of GPs at inclusion and when they ceased their activity, and between the characteristics of GPs at inclusion and the main criterion. All significant variables and the randomization arm were included in the model. The number of imputations performed corresponded to the percentage of data to be imputed. Multiple imputation was performed using chained equations and predictive mean matching (*mice* library).

A sensitivity analysis of the per-protocol population was also performed, excluding GPs who had ceased practice and those who had not received a visit from the HIR.

The overall volume of antibiotics dispensed at the end of the 12-month follow-up, per participating GP, was compared between arms using a 2-level hierarchical analysis of covariance (ANCOVA) adjusted for annual antibiotic prescription volume at baseline, with practice as a random effect.

The volumes of antibiotics dispensed after three months to patients considered critical (aged >65 years or <6 years) were also compared between groups using a 2-level hierarchical ANCOVA.

All tests were 2-tailed, with significance defined as *P*<.05. Model assumptions were verified. Analyses were performed using R software (version 3.6.0; Foundation for Statistical Computing).

### Ethical Considerations

Nantes University Hospital has adopted the MR004 Reference Methodology, which this protocol complies with. HIR visits are standard practice in the French health insurance system and seek to promote best practices regarding public health. The need to obtain consent from participating GPs and their patients was waived, according to rules governing the exceptional use of health data without the formal consent of participants [29]. Thus, GPs in the intervention groups were unaware that they had been involved in an intervention study, and GPs in the control group were not informed that their antibiotic prescription was monitored for the duration of the trial.

GP confidentiality was ensured with pseudonymized identifiers created by independent data managers of the regional division of the NHIS.

On July 18, 2019, the protocol (Multimedia Appendix 5) was approved under 110719107 by the Ethics Committee of the National College of Teaching General Practitioners (IRB00010804; Multimedia Appendix 6).

## Results

### Overview

**Figure 1:**
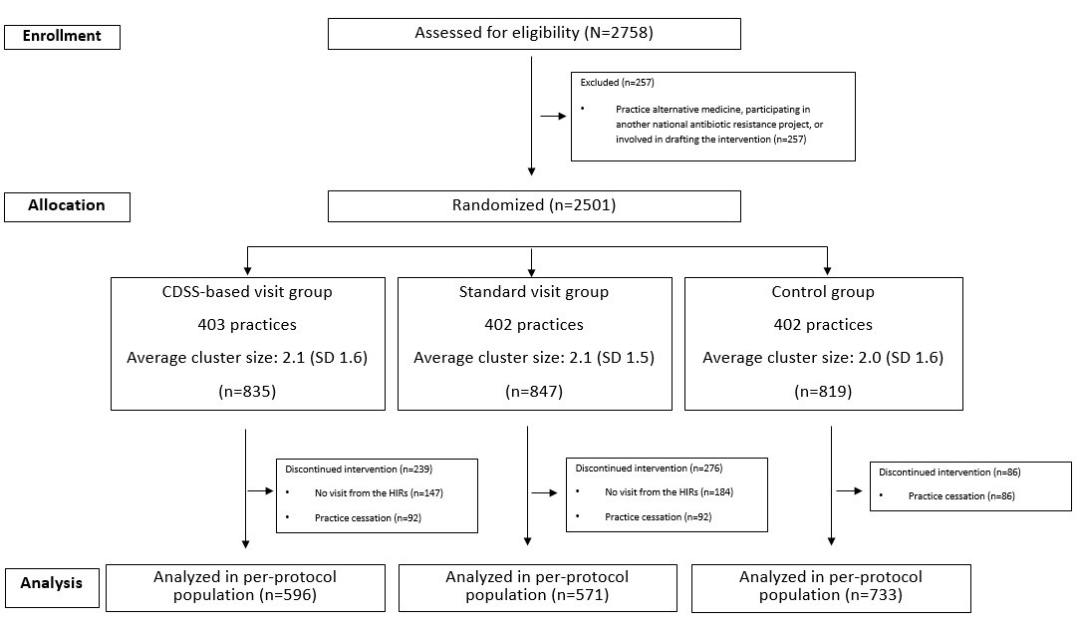
CONSORT (Consolidated Standards of Reporting Trials) flowchart. CDSS: clinical decision support system; HIR: health insurance representative.

Among the 2501 GPs included in the study, 835 were randomly assigned to the CDSS-based visit group, 847 to the standard visit group, and 819 to the control group (Figure 1).

Demographic, professional, and antibiotic prescribing characteristics of GPs are provided in Table 1.

**Figure 2:**
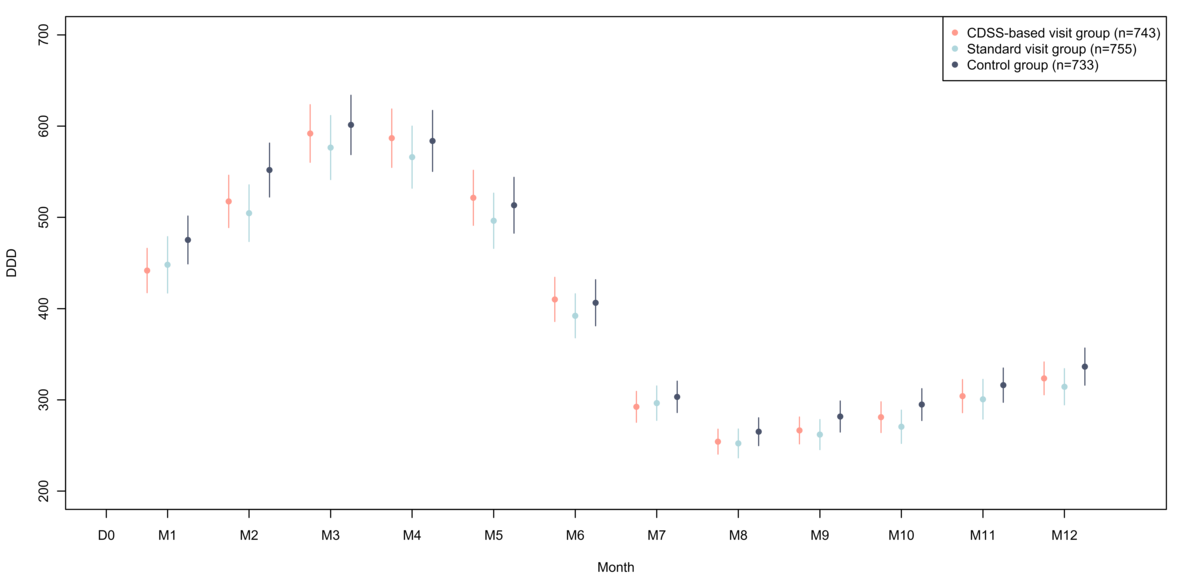
The mean volume of systemic antibiotics dispensed per month. Error bars indicate 95% CI. N is defined as general practitioners in continuous practice during the follow-up period. CDSS: clinical decision support system; D0: day 0 intervention; DDD: defined daily doses; M: month.

The monthly volume of antibiotics dispensed (DDD) per participating GP is shown in Figure 2.

**Table 1:** Baseline characteristics of participating general practitioners.

Interventions					CDSS^a^-based visit group (n=835)			Standard visit group (n=847)			Control group (n=819)
**Sociodemographic characteristics, n (%)**											
	**Age group (years)**										
		25-35	59 (7.0)			70 (8.3)			77 (9.4)		
		36-45	99 (11.9)			97 (11.4)			107 (13.1)		
		46-55	185 (22.2)			181 (21.4)			168 (20.5)		
		56-65	176 (21.1)			203 (24)			179 (21.9)		
		≥65	316 (37.8)			296 (35)			287 (35.1)		
		Not available	0 (0)			0 (0)			1 (0.1)		
	**Woman**			362 (43.3)			378 (44.6)			359 (43.8)	
**Workplace characteristics**											
	Group practitioner, n (%)			639 (76.5)			646 (76.3)			611 (74.6)	
	Sole practitioner, n (%)			196 (23.5)			201 (23.7)			208 (25.4)	
	Consultations per year, mean (SD)			4834.7 (1921)			4758 (2016)			4828.7 (1971)	
	Patients, mean (SD)			934 (424)			901.2 (438)			903.9 (436)	
	Activity ceased in the follow-up year, n (%)			92 (11)			92 (10.9)			86 (10.5)	
**Annual volume of systemic antibiotic prescriptions (DDD^b^) over the year preceding the start of the follow-up**											
	All, mean (SD)			5714.2 (3870.7)			5506.4 (4271.1)			5661.7 (3675.2)	
	Critical antibiotics, mean (SD)			1534.3 (1348.5)			1424.0 (1334.6)			1539.2 (1261.1)	
**Health Insurance Representatives visits conducted, n (%)**					648 (77.6)			621 (73.3)			—^c^

^a^CDSS: clinical decision support system.

^b^DDD: defined daily dose.

^c^Not applicable.

### Primary Outcome

At 12 months follow-up, the mean volume of systemic antibiotics per GP decreased by 219.2 (SD 61.4; 95% CI −339.5 to −98.8; *P*<.001) DDD in the CDSS-based visit group compared with the control group (Table 2).

The decrease in the mean volume of antibiotics dispensed per GP was not significantly different between the standard visit group and the control group (−109.7, SD 62.4; 95% CI −232.0 to 12.5 DDD; *P*=.08).

The per-protocol analysis is presented in Multimedia Appendix 7.

**Table 2:** Total volume of systemic antibiotics dispensed (DDD) per participating general practitioner (GP) at 12-month follow-up.

Arm	GPs in continuous practice during the follow-up period (available data), n	Total volume of systemic antibiotics dispensed, mean (SD)^a^	All GPs, n	Absolute difference (95% CI)^b^	Absolute standardized mean difference^b^	*P* values^b^
CDSS^c^-based visit group	743	4791 (3353.7)	835	−219.2 (−339.5 to −98.8)	−3.57	<.001
Standard visit group	755	4680 (3754.5)	847	−109.7 (−232.0 to12.5)	−1.76	.08
Control	733	4930 (3467.1)	819	Reference	Reference	Reference

^a^Based on available data.

^b^Based on all randomized GPs after multiple imputations. The total volume of systemic antibiotics dispensed was compared between arms using a 2-level hierarchical analysis of covariance adjusted for annual antibiotic prescription volume at baseline, with practice as a random effect.

^c^CDSS: clinical decision support system.

### Secondary Outcomes

**Figure 3:**
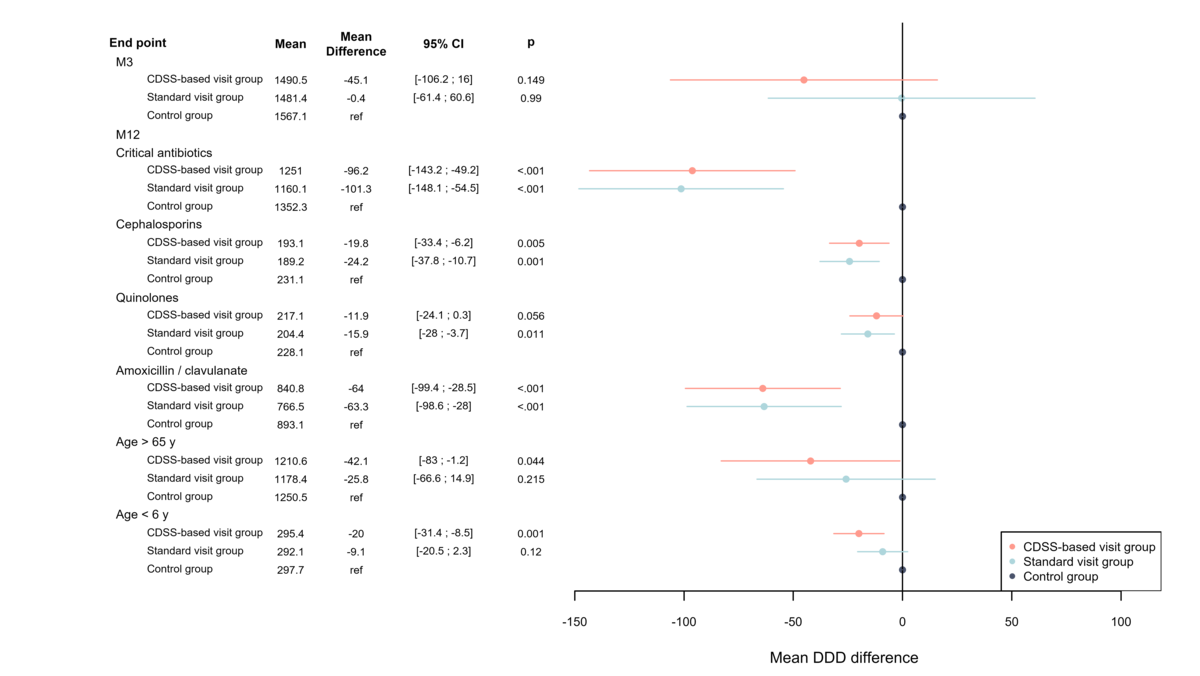
Subgroup analysis of the volumes delivered. Error bars indicate 95% CI. CDSS: clinical decision support system; DDD: defined daily doses; M: month.

A reduction in the volume of systemic antibiotic prescriptions was not observed at a 3-month follow-up for either group (Figure 3).

At 12 months, there was a reduction in both intervention groups (CDSS based and standard) in the volume of prescriptions for critical antibiotics, particularly cephalosporins, quinolones, and amoxicillin-clavulanic acid. In the CDSS-based group, there was also a reduction in the volume of prescriptions for patients aged >65 years and <6 years.

## Discussion

### Principal Findings

This study is the first randomized controlled trial to demonstrate the positive effect of an antibiotic stewardship intervention involving visits by HIRs, feedback, and the presentation of a CDSS dedicated to antibiotic prescribing at the population scale in France. Considering that France is the fourth-largest country in Europe in terms of antibiotic consumption [30], our findings, which show a 4.4% (-219.2/4930) reduction in the overall volume of consumption over 12 months, are very encouraging.

Our study shows that combining feedback, a face-to-face visit to the GP’s practice, and the promotion of an easy-to-use CDSS leads to a reduction in antibiotic prescribing in primary care. These results support and enrich those previously reported in the literature. For example, Ivers et al [31] and Daneman et al [10] showed the effectiveness of auditing and feedback in changing professional practices. Conversely, Aghlmandi et al [32] reported that audits and feedback had no effect. One reason for these conflicting results might be that their intervention involved email (mailing), while our intervention included an in-person HIR visit to GPs. The results of interventions involving the use of a CDSS have also been reported. In hospitals, Nachtigal et al [18] and Carracedo-Martinez et al [33] demonstrated better adherence to recommendations and reduced exposure to antibiotics through the use of a CDSS. In a systematic review targeting primary care, Holstiege et al [19] found a moderate impact of a CDSS on antibiotic prescribing. The effectiveness of our intervention is undoubtedly due to its multifaceted nature, which combines a CDSS demonstration with feedback.

It is likely that a HIR visit to the GP’s practice facilitates behavioral change, especially when combined with an easy-to-use CDSS, thus improving adherence to recommendations [20]. The success of the CDSS visit may also be related to the level of CDSS use in our region. Many GPs in the region were already CDSS users [34], and most knew how it worked, which probably made it easier to adopt. Our study suggests that these findings might be generalizable to the primary care setting. The positive effect of both interventions (an HIR visit, feedback, and either a CDSS demonstration or an information leaflet) on antibiotic prescribing (notably regarding broad-spectrum antibiotics and cephalosporins) is also relevant, and this result is consistent with the findings of Høgli et al [8]. The appropriate prescription of antibiotics is a vital issue in the context of increasing antibiotic resistance. The addition of the CDSS demonstration led to fewer antibiotics being prescribed in subgroups of the population that are highly exposed, notably patients aged >65 years and <6 years.

Finally, it should be noted that while, overall, the impact of the intervention was significant, this was not the case at 3 months, which suggests that there was a familiarization and learning effect regarding the CDSS. One hypothesis is that GPs gradually get into the habit of using the CDSS when they prescribe antibiotics for different infections and, thus, improve the overall quality of their prescriptions, in line with national recommendations. A learning effect has already been described when using Antibioclic in clinical studies [23]. It reported no effect on prescriptions from a recently used computer program. The success of the CDSS visit may also be related to the level of CDSS use in our region. The GPs in the region were already heavy CDSS users [34], and most knew how it worked, which probably made it easier to adopt.

A future study in France could examine how the CDSS is used by the biggest prescribers of antibiotics. In Canada, Schwartz et al [35] have shown that an intervention with this type of prescriber can be effective.

### Strengths and Weaknesses

Our study has various strengths. Its pragmatic design allowed the intervention to be evaluated under real-life conditions. As a regional project, it involved a large number of GPs. The data sources used to measure outcomes were robust, reliable health care administration databases. Considerable thought and care went into designing this study, and it is in line with recent recommendations on antimicrobial stewardship intervention evaluation [36]. The impact of the intervention, in terms of better practices, is likely to go beyond the 2 pathologies studied here, as the Antibioclic CDSS can be applied to other pathologies. We know, for example, that over 50% of CDSS queries concern 6 pathologies, including cystitis and tonsilitis [21]. The effect on practices may therefore have been underestimated. Our findings are interesting, as there is no consensus in the literature regarding the positive effects of multimodal computerized interventions [37].

Our study also has various limitations. We have no data on the implementation, or actual use of the CDSS by GPs, as this was not measurable. Furthermore, clinical indications for antibiotic prescriptions are not available in the SNIIRAM database. It is therefore impossible to conclude that there may be an improvement in appropriate prescriptions. In addition, it would have been interesting to assess the sustainability of the effect of the intervention beyond 12 months. However, cost and feasibility limitations meant that we were unable to carry out the necessary follow-up. Finally, even if the intervention can be easily reproduced on a national scale since HIR visits are routine practice in all regions in France and are managed by the NHIS, the generalization of our findings to the international context would require the creation of HIRs in other health insurance systems.

Although the study design is based on a randomized controlled trial, it is possible that the periods of lockdown related to the COVID-19 pandemic, beginning on March 17, 2020, could have affected our results. During this period, there was a historic decrease (17%) in antibiotic consumption in France [27]. While antibiotic prescription volumes decreased significantly in each of our 3 groups, this decrease was greater in the CDSS and standard visit groups than in the control group. The gap between the arms may have been even greater if the study had not been conducted during the pandemic. Consequently, it would be interesting to replicate the study in a nonpandemic context.

### Conclusion

Our study found that the combination of a HIR visit and feedback, together with a presentation of the CDSS Antibioclic, led to a 4.4% (-219.2/4930) reduction in the volume of systemic antibiotics prescribed after 12 months. In addition, visits that provided feedback, both with and without the presentation of the CDSS, led to a reduction in the volume of critical antibiotics (broad-spectrum and cephalosporins) prescriptions.

## Appendix

Multimedia Appendix 1 CONSORT (Consolidated Standards of Reporting Trials) checklist.

Multimedia Appendix 2 Tonsilitis, choice of criteria.

Multimedia Appendix 3 Treatment of tonsillitis.

Multimedia Appendix 4 Leaflet used in the “standard visit group” intervention.

Multimedia Appendix 5 Research protocol.

Multimedia Appendix 6 Ethical approval.

Multimedia Appendix 7 Total volume of systemic antibiotics dispensed in defined daily doses (DDD) per participating general practitioner after 12-month follow-up (per-protocol analysis).

## Abbreviations

ANCOVA: analysis of covariance
CDSS: clinical decision support system
CONSORT: Consolidated Standards of Reporting Trials
DDD: defined daily doses
GP: general practitioner
HIR: health insurance representative
NHIS: National Health Care Insurance Organization
SNIIRAM: Système National d’Information Inter-Régimes de l’Assurance Maladie

## Data Availability

All data generated or analyzed during this study are included in this published article and its supplementary information files.
