# Supplementary figures and images for: Effect of a Feedback Visit and a Clinical Decision Support System Based on Antibiotic Prescription Audit in Primary Care: Multiarm Cluster-Randomized Controlled Trial
Source: J Med Internet Res. 2024 Dec 18;26:e60535. doi: 10.2196/60535 (PMC11694052; doi:10.2196/60535)

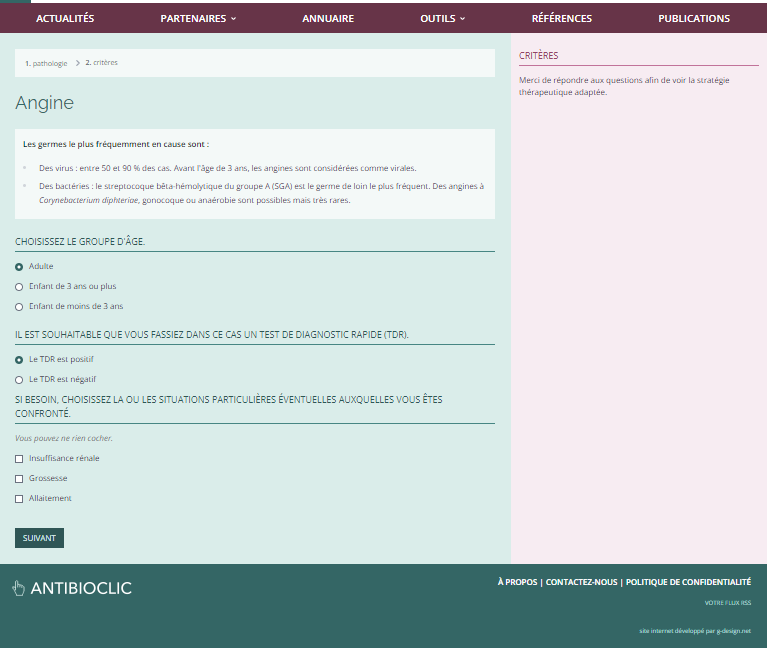

Supplement: Multimedia Appendix 2 [file jmir_v26i1e60535_app2.png]

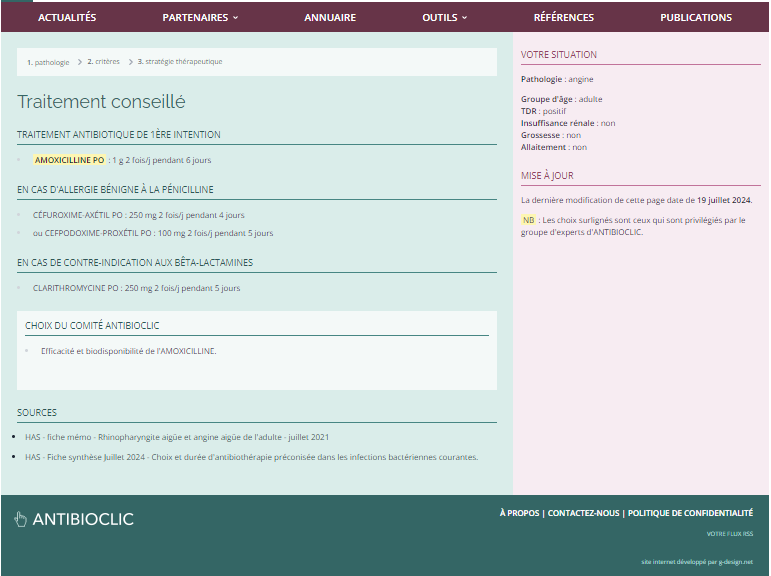

Supplement: Multimedia Appendix 3 [file jmir_v26i1e60535_app3.png]

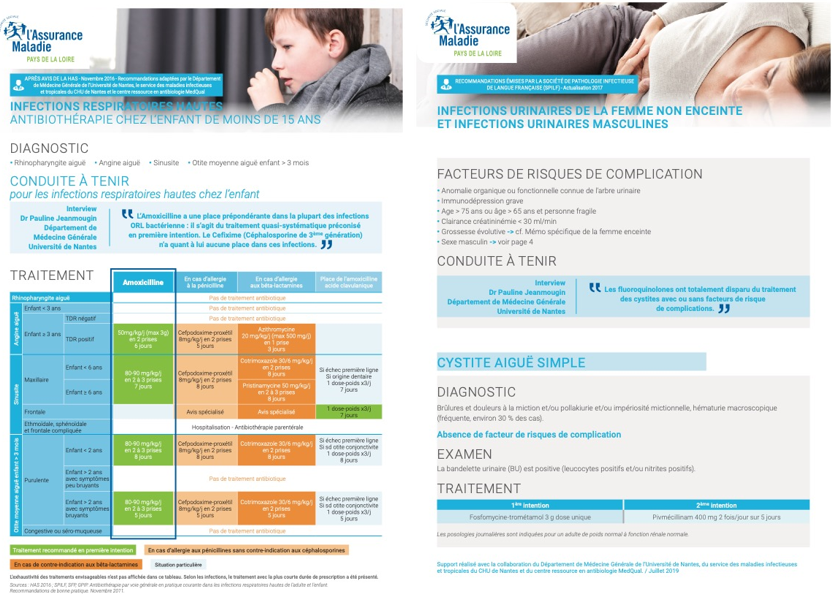

Supplement: Multimedia Appendix 4 [file jmir_v26i1e60535_app4.png]
