# Supplementary material for: Effect of a Feedback Visit and a Clinical Decision Support System Based on Antibiotic Prescription Audit in Primary Care: Multiarm Cluster-Randomized Controlled Trial
Source: J Med Internet Res. 2024 Dec 18;26:e60535. doi: 10.2196/60535 (PMC11694052; doi:10.2196/60535)
Supplement: Multimedia Appendix 5 [file jmir_v26i1e60535_app5.docx]

***ACTION***

***Antibiotic - primary Care - Intervention***

**« Impact on antibiotic prescriptions of a bundle intervention conducted by medical representatives in general practitioner facilities, based on operational demonstration of an Internet decision support tool: Antibioclic »**

RESEARCH PROTOCOL NOT INVOLVING THE HUMAN PERSON

**Principal investigators :**

**Dr Stéphanie LARRAMENDY and Pauline JEANMOUGIN**

Department of General Medicine, University of Nantes

1 rue Gaston Veil - 44000 NANTES

Tel. 02.40.41.28.28 - Fax 02.40.82.67.76

**Coordinator in charge of methodology and scientific development :**

**Dr Cédric RAT**

Department of General Medicine, University of Nantes

1 rue Gaston Veil - 44000 NANTES

Tel. 06 61 86 79 53 - Fax 02.40.82.67.76

[*cedric.rat@univ-nantes.fr*](mailto:cedric.rat@univ-nantes.fr)

**Promoter :**


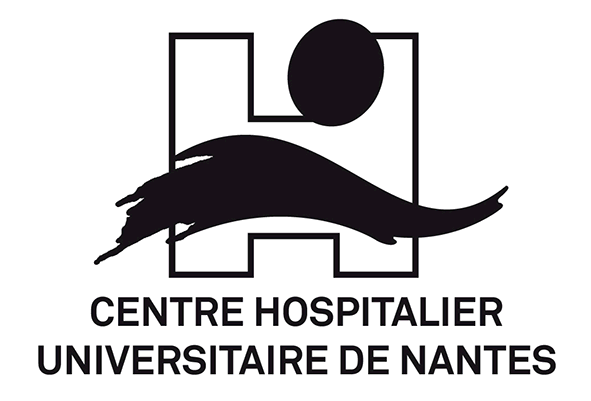
**Nantes University Hospital**


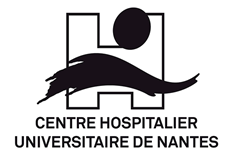
Medical Affairs Department

et de la Recherche
5, allée de l'île Gloriette
44 093 Nantes cedex 01 (FRANCE)

Tel: 02 53 48 28 35
Fax : 02 53 48 28 36

In partnership with the CPAM, the Nantes CHU infectious and tropical diseases department, ANTIBIOCLIC and MEDQUAL

**LIST OF ABBREVIATIONS**

| ADV | Visual Help |
| --- | --- |
| ANSM | French National Agency for the Safety of Medicines |
| ATB | Antibiotic |
| ATC | Anatomical, Therapeutic and Chemical Classification |
| CEREES | Expertise Committee for Research, Studies and Assessments in the field of Health |
| CHU | University Hospital |
| CNIL | Commission Nationale de l'Informatique et des Libertés (French Data Protection Authority) |
| CPAM | Caisse Primaire d'Assurance Maladie |
| DAM | Health Insurance delegate |
| DDJ | Defined Daily Dose |
| ITT | Intention to treat |
| MG | General Practitioner |
| WHO | World Health Organisation |
| ENT | Ear, Nose and Throat |
| RNI | Non-interventional research |
| SNDS | National Health Data System |
| SNIIRAM | National Health Insurance Inter-Scheme Information System |
| WHO | World Health Organization |
|  |  |

1. **RATIONAL OF THE STUDY**

The loss of antibiotic efficacy is a threat to public health [1-4]. The mechanisms behind antibiotic resistance are multiple and complex, but one of the main mechanisms is the high and excessive consumption of antibiotics [5-8]. France is the 4th largest consumer of antibiotics in Europe [9-10]. Between 2011 and 2016, antibiotic consumption in towns and cities rose by 5.6% [10]. In France, as in other countries, the majority of prescriptions are made by GPs. In France, general practitioners account for 70% of these prescriptions. Several studies suggest that non-clinical factors influence these prescriptions, which suggests possible areas for improvement [11,12], particularly in paediatrics [13]. It would therefore seem vital to implement initiatives specifically aimed at GPs.

Numerous experiments have been carried out internationally with a view to changing antibiotic prescribing practices among GPs [14-17]. Simply developing training courses or making information available on the internet does not appear to change practices if these interventions are carried out in isolation [3]. On the other hand, various approaches involving communication training, specific educational interventions based on examples of preconceived ideas, interventions at the point of care, and the use of electronic decision support systems have been shown to have beneficial effects on prescribing [14-27]. Attali et al, in the PAAIR study, showed the importance of working on the preconceived ideas that GPs may have [25]. Giving GPs feedback on their prescribing practices has also been shown to have an impact [14;20;28-30]. Many authors conclude that multifaceted interventions are needed [22]. The case of paediatric prescribing appears to be particularly difficult [31-34]. In a recent study, Gulliford et al. were able to demonstrate that feedback on practices can improve antibiotic prescribing practices in the general population, but this intervention had no impact in paediatrics, or in people over 85 years of age [20].

In France, the Antibioclic website was created in 2011 by a multidisciplinary committee of GPs and infectious diseases specialists from universities and hospitals at the University of Paris Diderot [35,36]. It is a web-based Clinical Decision Support System developed for GPs, which can be accessed directly, freely and at no cost, without the need for identification or registration. It provides advice on prescribing antibiotics for all infectious diseases commonly encountered in primary care, based on current national recommendations. A decision-making algorithm is proposed for 40 infectious diseases encountered in ambulatory care. The originality of the tool lies in its user-friendliness and its ability to propose, in a simple format, a course of action that takes account of patient characteristics: age (child/adult), identification of chronic renal failure, pregnancy or breast-feeding, for example. The Antibioclic committee monitors the literature and adapts the content of the tool as new recommendations are published. Antibioclic has met with growing success since its launch: around 9,500 users log on every day (Google Analytics data^®^ ), making more than 2 million requests a year. In a 2014 cross-sectional survey of 1,344 users, 90% were identified as general practitioners [37].

A number of studies suggest that doctors who use Antibioclic have different antibiotic prescribing practices to those who do not. In 2016, Heslot C. et al. surveyed 142 general practitioners in Isère to assess the median duration of amoxicillin prescriptions for acute community-acquired pneumonia. In this survey, 79% of respondents stated that they used Antibioclic to obtain information on antibiotic therapy, whereas only 58% consulted national recommendations, 52% consulted specialist medical journals and 34% consulted the website of the Société de pathologie infectieuse de langue française (SPILF) [38]. Only doctors who reported consulting Antibioclic had a prescription duration in line with the 7-day treatment recommendation [38]. In the Aquitaine region, a prospective study conducted between 25 October 2016 and 3 February 2017 at the Libourne Hospital Centre among 19 GPs found that, out of a total of 136 antibiotic therapy prescriptions, those initiated during a consultation using the Antibioclic tool had a duration more in line with national recommendations, and the choice of molecules used appeared to be more appropriate [39]. One limitation of these studies is that they do not allow conclusions to be drawn about the impact of using the site on practices, or whether using the Antibioclic site is simply a marker for identifying doctors who are more aware (from the outset) of the need to prescribe antibiotics correctly. While some authors suggest that the use of online prescribing aids could help to reduce antibiotic prescribing by doctors [40-41], the Antibioclic website is only used by a minority of professionals.

With a view to optimising prescriptions, our idea for this project is to implement a multi-faceted strategy that can be rolled out nationwide, combining different intervention methods that have demonstrated their impact, and to involve the use of the Antibioclic Clinical Decision Support System. The proposed study will therefore test a multifaceted intervention method based on 1) a visit by a medical representative to doctors' surgeries, with: raising awareness of antimicrobial resistance, work on preconceived ideas as in the PAAIR study, feedback on prescriptions, AND 2) use of the Antibioclic Clinical Decision Support System.

The generalisability of the intervention will be based on collaboration with the Délégués de l'Assurance Maladie (DAM), who are already working on this issue on an operational and regular basis throughout France [42]. The DAMs, who are based throughout the country, make regular visits to GPs and promote good practice [43]. As this method of visiting GPs is original on an international scale, it is vital to demonstrate its impact on practices.

**The aim of the research** is to evaluate the effect of multifaceted interventions on GP prescribing by comparing the volumes of antibiotics dispensed at the end of 12 months of follow-up in a study comparing :

i) an intervention carried out by the Délégués d'Assurance Maladie (health insurance representatives) in general practices, combining a usual visit (with awareness-raising on antibiotic resistance, work on preconceived ideas, feedback on practices) and a demonstration of the use of Antibioclic (arm 1),

ii) an intervention carried out in the same way by Health Insurance delegates but without demonstration of Antibioclic (arm 2),

iii) compared with usual practice (arm 3).

1. **ORIGINALITY AND INNOVATION**

The project proposes an original study based on collaboration between various primary care players: a university team of general practitioners, regional health insurance teams in Pays de la Loire, a university hospital infectiology team, the MedQual centre (Antibiotic Resource Centre for the Pays de la Loire region) and the Antibioclic Clinical Decision Support System team.

The project was co-constructed with the Pays de la Loire health insurance scheme.

The originality of the intervention lies in the approach combining the strengths of tools that already exist and are established, but which act without synergy, despite their strong potential added value: face-to-face interventions by Assurance Maladie delegates in doctors' surgeries, support materials (enabling feedback on practices) built from prescription data collected by Assurance Maladie, and the Antibioclic internet tool to help with prescribing.

The robust design of a randomised study in 3 parallel arms covering the entire population of GPs in an administrative region will enable us to conclude both on the effect of a specific intervention by health insurance delegates in the form of the usual visits implemented by the social security system, and on the specific added value of demonstrating the use of Antibioclic.

The proposed intervention is replicable on a national scale and could serve as a model for the future.

The method chosen for data collection, based on collaboration with health insurance departments and extraction of data from the SNDS, will help to limit missing data.

1. **DESCRIPTION OF EXPECTED BENEFITS**

The aim of the study is to identify the most effective ways of reducing the volume of antibiotics dispensed, with immediate benefits for public health:

. limiting the development of bacterial resistance,

. optimising antibiotic prescriptions (better quality prescriptions),

. lower costs.

The results will provide original information, at the end of a randomised comparative study, on the specific impact of 2 complementary types of intervention:

1. the use of visits by health insurance delegates,

2. demonstration of the use of the Antibioclic site.

Secondary analyses will enable us to better characterise the differentiated impact of the intervention according to prescribers or patient characteristics.

The study will provide an opportunity to raise GPs' awareness of the problem of antibiotic resistance on a large scale, and will raise awareness of :

. more than 800 doctors included in arm 1, who will receive awareness training from the Health Insurance delegate, feedback on their practices, and a demonstration of how to use the Antibioclic website,

. more than 800 doctors included in arm 2, who will benefit from awareness-raising by the Health Insurance Delegate and feedback on their practices.

The medico-economic study will document the potential impact on healthcare expenditure.

One of the ancillary benefits of the study will be to mobilise the partners around a joint project, on a theme that is of daily interest to primary care professionals.

1. **OBJECTIVES AND ASSESSMENT CRITERIA**
   1. ***OBJECTIVE AND MAIN EVALUATION CRITERIA***

***4.1.1. Main objective***

The main objective is to compare the effect on antibiotic dispensing after 12 months of follow-up,

i) an intervention carried out by the Délégués d'Assurance Maladie in general practices, combining a routine visit (raising awareness of antimicrobial resistance, work on preconceived ideas, feedback on practices) and a demonstration of the use of Antibioclic (arm 1),

ii) an intervention carried out in the same way by the Health Insurance delegates, but without Antibioclic being demonstrated (arm 2),

iii) compared to usual practice (arm 3). In the latter arm, the Health Insurance delegates carry out a visit that does NOT involve prescribing antibiotics.

***4.1.2. Primary endpoint***

The primary endpoint will be the overall volume of antibiotics dispensed in defined daily doses (DDD) per participating doctor, at the end of 12 months' follow-up.

Antibiotics used systemically (oral, parenteral, ATC class J01) will be taken into account. Antibiotics used topically by the cutaneous, ocular or auricular route will be excluded.

The DDDs will be calculated by the regional branch of the Assurance Maladie. The method for calculating DDDs will be that used by the World Health Organisation [44,45]. The exhaustiveness of the Assurance Maladie database will limit the risk of measurement bias.

NB: Antibiotic prescriptions are reported to the GP if they follow a prescription bearing the GP's ADELI / RPPS number.

- 1. ***OBJECTIVES AND SECONDARY EVALUATION CRITERIA***
     1. ***secondary objectives***

1/ Analyse the effect on the volumes of antibiotics delivered over time, in each of the 3 arms,

. by comparing the volumes of antibiotics dispensed at the end of 3 months' follow-up,

. by describing monthly changes in the volumes of antibiotics dispensed, over the entire monitoring period (12 months).

The monitoring period for the doctor's prescriptions will begin from the date of the visit by the Health Insurance delegate (T0). (See also Chapter 6.3. Inclusion and follow-up period).

2/ To analyse the specific effect on the volumes of broad-spectrum antibiotics most frequently responsible for antibiotic resistance in primary care (3rd generation cephalosporins, fluoroquinolones, amoxicillin/clavulanic acid) dispensed [8] [9] in each of the 3 arms.

. by comparing the volumes of antibiotics dispensed at the end of 12 months of follow-up,

. by describing monthly changes in the volumes of antibiotics dispensed over the entire monitoring period (12 months).

3/ To analyse the effect on volumes of antibiotics dispensed in two specific patient subgroups: patients aged over 65 (subgroup 1), and children under 6 (subgroup 2), in each of the 3 arms.

. by comparing the volumes of antibiotics dispensed at the end of 12 months of follow-up,

. by describing monthly changes in the volumes of antibiotics dispensed, over the entire monitoring period (12 months).

4/ Analyse the factors associated with the dispensing of antibiotics :

The demographic characteristics (age, sex) of the doctors and their mode of practice (type of commune of practice based on INSEE urban area zoning, "major centres" (codes 111, 112, 120), "small centres" (codes 211, 212, 221, 222), "isolated towns" (code 400), and "other communes" (code 300) [46]), group practice/independent practice, number of procedures/year, number of patients declared as attending physician) will be evaluated as factors potentially associated with the volume of antibiotics dispensed at the end of 12 months of follow-up.

5/ Compare the efficiency of drug dispensing after 12 months of follow-up.

- - 1. ***Secondary evaluation criteria***

The secondary evaluation criteria will be as follows:

1/ Overall volume of antibiotics dispensed at the end of 3 months' follow-up, measured in DDD per participating doctor.

Overall monthly volume of antibiotics dispensed, measured in DDD per participating doctor.

2/ Volume of broad-spectrum antibiotics most frequently responsible for antibiotic resistance in primary care (3rd generation cephalosporins, fluoroquinolones, amoxicillin/clavulanic acid) dispensed, measured in DDD per participating doctor.

3/ Sub-group analyses :

. Volume of antibiotics dispensed to patients over 65 (sub-group 1), measured in DDDs per participating doctor,

. Volume of antibiotics dispensed to children under 6 (sub-group 2), measured in DDD per participating doctor.

4/ Analysis of physician factors associated with the overall dispensing of antibiotics: age, sex, type of practice municipality [46], group practice/independent practice, number of procedures performed during the 12 months of follow-up, total number of patients declared in the attending physician's patient base.

5/ Cost-effectiveness analysis :

Incremental cost-effectiveness ratio comparing the 3 alternatives: i) DAM visit with Antibioclic demonstration, ii) DAM visit without Antibioclic demonstration, iii) usual practice.

1. **STUDY POPULATION**
   1. ***Description of the population***

The study covers the 5 departments of the Pays de la Loire region.

All GPs practising in the region who have seen more than 100 different patients in the year preceding the evaluation will potentially be eligible. The list of eligible GPs in the 5 departments will be established through a partnership with the Assurance Maladie, as in a previous study conducted by our team [47]. A preliminary study will enable us to anticipate the inclusion of more than 2,400 doctors.

- 1. ***Inclusion criteria***

GPs practising in one of the 5 study departments who had seen at least 100 different patients (of any age) in the year preceding the evaluation.

- 1. ***Non-inclusion criteria***

Not included:

. doctors with special practices (acupuncture, allergology, angiology). These doctors will be identified on the basis of health insurance data.

. doctors included in another programme to improve antibiotic prescribing practices run by the Assurance Maladie in the form of a "Plan Personnalisé d'Accompagnement" (CPAM initiative).

. doctors who participated in the design of the study: doctors who took part in the steering groups and working groups, doctors who tested the content of the DAM visit.

1. **DESIGN AND CONDUCT OF THE STUDY**
   1. ***General research methodology***

. This will be a prospective, randomised, controlled, open-label study with 3 parallel arms.

. The study will involve all GPs in the 5 departments of the Pays de la Loire region who meet the inclusion and non-inclusion criteria.

. The statistical unit will be the doctor.

. The source of the data will be the Health Insurance SNDS database for the Pays de la Loire region.

. Randomisation will be based on doctors' surgeries (and not doctors) to limit confounding bias between arms.

. The analysis will be carried out on an intention-to-treat basis.

This is a 'real-life' study.

In particular, it will not be a question of evaluating the effectiveness of the Antibioclic site "*per* protocol".

The challenge will be to assess the effect of the 2 interventions, compared with usual practice, bearing in mind that the Health Insurance Representatives' visit and its potential impact will vary greatly from one doctor to another.

- 1. ***RANDOMISATION; INTERVENTION ARMS and CONTROL ARMS***

**At the end of randomisation, participating doctors will be randomly assigned to one of the following 3 arms:**

**Arm 1: Presentation by the DAM with demonstration of the ANTIBIOCLIC internet tool**

. Raising awareness of antibiotic resistance, highlighting the classes of antibiotics that are sources of resistance,

. A look back at prescribing practices, with a presentation of personal data from the Assurance Maladie,

. Deconstruction of preconceived ideas and strategies for resolving high-risk situations (based on the PAAIR study [23],

. Demonstrating the use of Antibioclic.

The presentation will focus on 2 common pathologies, to deliver key messages: 1) cystitis (message: no place for fluoroquinolones since the SPILF [48] updated its recommendations), 2) bacterial angina (message: amoxicillin alone, very little place for amoxicillin-clavulanic acid in ENT infections). Doctors use Antibioclic by browsing the site themselves. They are guided by the Health Insurance delegate. The intervention includes the installation of the link in favourites, and an incentive to use the site again in the future (see also the description of the DAM intervention in 5.4.).

**Arm 2: DAM intervention without demonstration of the ANTIBIOCLIC internet tool**

. Raising awareness of antibiotic resistance, highlighting the classes of antibiotics that are sources of resistance,

. A look back at prescribing practices, with a presentation of personal data from the Assurance Maladie,

. Deconstructing preconceived ideas and strategies for resolving high-risk situations,

. Presentation and distribution of paper memos on 2 common pathologies, and delivery of key messages: 1) cystitis (message: no place for fluoroquinolones since the SPILF [48] updated its recommendations), 2) bacterial angina (message: amoxicillin alone, very little place for amoxicillin-clavulanic acid in ENT infections).

**Arm 3. Usual practice (without intervention relating to antibiotic prescribing)**

. Presentation of a different theme (not relating to antibiotic prescribing or antibiotic resistance) during the Health Insurance representative's visit to the doctor.

- 1. ***Creation of the necessary support materials***

The various partners in the project will work together to create the materials required for the 2 intervention arms: the Department of General Medicine at the University of Nantes, the Pays de la Loire Health Insurance Scheme - including representatives of the health insurance delegates - and the antibiotic referral agents at Nantes University Hospital.

The project includes a number of preparatory meetings to develop the materials needed for the DAMs' work. These materials will be essential for standardising the visit and the messages delivered in the two arms presenting the antibiotics theme.

- The work of the Health Insurance Representatives will be based on a digital visual aid, a slide show on an I-Pad-type tablet usually used by the DAMs when visiting doctors.

The digital visual aid will successively review epidemiological data on antibiotic resistance and 3 major factors in bacterial resistance (wrong indication, wrong duration, wrong molecule).

This will be followed by a discussion of practical solutions to these 3 causes of overconsumption: presentation of Antibioclic (in arm 1), or distribution of memo cards (in arm 2),

Lastly, the support will lead to a discussion of preconceived ideas about antibiotics, and will propose strategies to help patients avoid prescribing, based on the PAAIR study [25].

With a view to training the DAMs, the study includes the drafting of a detailed sales pitch corresponding to the elements included in the digital visual aid. Exploring in greater depth all the information contained in the digital visual aid should enable DAMs to feel at ease with the subject, to be aware of the specific issues involved in prescribing antibiotics in primary care, and to be able to answer any questions from the doctors visited.

- The project involves the production of fact sheets based on the CNAM fact sheets on upper respiratory infections and urinary tract infections:

. Upper respiratory infections :

The plan is to simplify and prioritise the information in the table of treatments on the initial CNAM form: amoxicillin will be given priority over other molecules, a single 2^e^ generation cephalosporin will be proposed in cases of allergy to penicillins (the one with the shortest duration of treatment), and amoxicillin-clavulanic acid will be relegated to exceptional use.

. Urinary tract infections: acute cystitis in non-pregnant women and men and acute cystitis in pregnant women.

The project involves updating the CNAM memo sheet with the latest SPILF recommendations from 2017 [48] instead of the 2015 HAS recommendations.

- 1. ***F*** ***ormation DAM***

Training for DAMs will be developed in collaboration with the various partners in the project: the Department of General Medicine at the University of Nantes, the Pays de la Loire health insurance scheme, involving representatives of the health insurance delegates, and the antibiotic referral agents at Nantes University Hospital.

The project is planning several preparatory meetings to set up training for the DAMs. The training of the DAMs will be essential in order to standardise the visit and the messages delivered in the two arms presenting the antibiotic theme.

- - 1. ***Creation of role plays for training DAMs***

The project involves the creation of role-playing games to enable the DAMs to take ownership of the discussion sessions with GPs. This type of role-playing session is customary in the training of DAMs prior to their work with GPs.

One of the role-plays will be built around a profile of a doctor with a higher rate of prescribing amoxicillin and clavulanic acid than the departmental average.

The second role-play will be built around a profile of a doctor with a higher rate of fluoroquinolone prescribing than the departmental average.

- - 1. ***Course of training for DAMs***

DAM training must include :

- presentation of the study protocol, emphasising the need to ensure random allocation of doctors to the 3 arms.
- presentation of the digital visual aid on the I-Pad tablet, which will be used by the DAMs during visits by doctors.

The presentation will review the epidemiological data on antibiotic resistance and the 3 major factors in bacterial resistance (wrong indication, wrong duration, wrong molecule).

The presentation will describe the practical solutions proposed in response to these 3 causes of overconsumption: presentation of antibioclic (arm 1) or distribution of memo cards (arm 2), discussion of preconceived ideas about antibiotics, and presentation of strategies to help avoid prescribing resulting from the PAAIR study [25].

- the presentation of detailed arguments, so that the DAMs are at ease with the subject, are aware of the specific problems of antibiotic prescribing in primary care, and can answer any questions from the doctors visited.
- presentation of CNAM memo sheets on upper respiratory infections and urinary tract infections. (
  1. ***Inclusions and follow-up period***

The period during which the doctor's prescriptions will be monitored will begin on the date of the DAM's visit (T0). For doctors in arms 1 and 2, the DAM's visit will be staggered over approximately 3 months, corresponding to the time usually required for DAMs to visit all the doctors in their intervention zone. For doctors in arm 3, the date of the DAM's visit will be noted even though the DAM will not be making a specific intervention relating to the prescription of antibiotics (presentation of a different topic during the visit by the Health Insurance Representative to the doctor). Monitoring will last 12 months from the date of the DAM's visit.

- 1. ***Study timetable***

M - 3: Health Insurance Representatives will be trained 3 months before the start of the first visits to doctors.

**T0:** Start of DAM visits to GPs.

M3: End of the cycle of visits made by the DAMs to GPs as part of the study.

Monitoring period*:* The doctor's prescriptions will be monitored from the date of the DAM's visit to the GP (T0). However, the DAMs' visits will be staggered over approximately 3 months, corresponding to the time usually required for the DAMs to visit all the doctors in their area of intervention. For doctors in arm 3, the date of the DAM's visit will be noted even though the DAM will not be making a specific intervention relating to the prescription of antibiotics (presentation of a different topic during the visit by the Health Insurance Representative to the doctor).

*NB: we are anticipating a potential additional 15-day period, beyond the 3-month period initially planned, in order to anticipate some postponements of appointments when doctors are on holiday.*

Monitoring will last 12 months from the date of the DAM's visit.

M15 (+ 15 days): End of the monitoring period corresponding to the last doctors visited.

1. **DATA MANAGEMENT AND STATISTICS**
   1. ***extraction of study data***
      1. ***DATA COLLECTION, PROCESSING AND CIRCULATION***

**Initial collection**

The Assurance Maladie will be responsible for extracting the data specific to the research and for building up the database.

The data collected will be data characterising GPs: age group (in 5-year increments from the age of 25), sex, classification of the commune of practice (according to INSEE urban area zoning, "major centres" (codes 111, 112, 120), "small centres" (codes 211, 212, 221, 222), "isolated towns" (code 400), and "other communes" (code 300) [46]), activity in terms of number of annual consultations, number of patients in the GP patient base, mode of practice (alone, in a group).

The Assurance Maladie randomises the GPs' places of practice and adds the allocated randomisation arm to the list of participating GPs, according to their place of practice.

The Assurance Maladie extracts data on antibiotic therapy prescriptions from all GPs included in the study (data for the 12 months preceding the visit) for descriptive purposes (T0).

**A 2nd round of** data **collection** will take place at the end of 12 months' follow-up: the French National Health Insurance will extract data on the dispensing of antibiotics prescribed by all GPs included in the study.

The data collection period will begin on the day of the DAM's visit.

- - 1. ***PARTICIPANT IDENTIFICATION***

The data will be centralised by the Assurance Maladie. No nominative data will be exported outside the databases of the Sickness Insurance departments.

The variables were selected after a pilot study, and the health insurance authorities paid close attention to CNIL regulations, to ensure that no data was directly identifiable (which is why the ages of practitioners are grouped into 5-year classes, and the communes classified by type).

- 1. ***Statistics***
     1. ***DESCRIPTION OF PLANNED STATISTICAL METHODS, INCLUDING TIMETABLE FOR INTERIM ANALYSES***

The analyses will be carried out using R 3.6.0 software.

The data will be described by the numbers and percentages of each modality for the qualitative variables and by the minimum, maximum, quartile, mean and standard deviation for the quantitative variables, overall and according to the 3 randomisation arms.

A hierarchical procedure will be used to successively test the comparison between the 3 arms, while maintaining a 5% alpha risk. The first hypothesis tested will be the difference between arm 1 and arm 3. If the test is significant, a second test will be performed comparing arm 1 to arm 2.

- - - 1. *Primary endpoint*

The overall volume of systemic antibiotics dispensed in DDD at the end of 12 months of follow-up, per participating doctor, will be calculated and compared between arms using a Student's t test.

- - - 1. *Secondary endpoints*

In addition to the primary endpoint (assessing the overall volume of systemic antibiotics dispensed at 12 months follow-up), other endpoints will be studied.

1/ The overall volume of systemic antibiotics dispensed in DDD at the end of 3 months of follow-up, per participating physician, will be calculated and compared between arms using a Student's t test. The monthly change in the overall volume of systemic antibiotics dispensed will also be described between arms.

2/ The volume of broad-spectrum systemic antibiotics most frequently responsible for antibiotic resistance in primary care (3^ème^ generation cephalosporins, fluoroquinolones, amoxicillin/clavulanic acid) dispensed in DDD per participating doctor will be calculated and described monthly and over 12 months of follow-up, and for the latter, compared between arms using a Student's t test.

3/ Sub-group analyses :

. Subgroup 1: The volume of systemic antibiotics dispensed to patients aged over 65 in DDD at the end of 12 months of follow-up, per participating doctor, will be calculated and compared between arms using a Student's t test.

. Subgroup 2: the same analysis will be carried out for patients under 6 years of age, and for these same patients on the broad-spectrum systemic antibiotics most frequently responsible for antibiotic resistance in primary care.

The monthly trends for these two sub-groups will also be described according to the randomisation arms.

The overall monthly volume of systemic antibiotics dispensed in DDDs, and by subgroup, will be described for each of the three groups, and represented graphically.

4/ The characteristics of the physicians (age, sex, type of practice, etc.), which may be associated with the overall prescription of systemic antibiotics, will also be investigated using ANCOVA models, adjusted for the randomisation arm.

5/ The secondary endpoints used for the medico-economic analysis are described in section 9. Medico-economic analysis, and in particular chapters 9.2, 9.3 and 9.4.

. The cost-effectiveness analysis will be conducted on the intention-to-treat (ITT) population, i.e. on all randomised healthcare professionals.

. Details of the resources consumed in each arm (averages and dispersion) and the average costs and efficiency per doctor will be calculated for each arm.

. A table will show the difference between the two groups in terms of costs and effectiveness, and the cost-effectiveness ratio will be calculated.

. As costs are not generally distributed according to a normal distribution, the incremental cost-effectiveness ratio and its confidence interval will be estimated using the non-parametric bootstrap method [49].

Finally, the probability that the use of antibioclic associated with a specific DAM visit is cost-effective compared with *a standard DAM visit* will be calculated for different values of willingness to pay for a reduction in antibiotics.

- - 1. ***STATISTICAL JUSTIFICATION FOR THE NUMBER OF INCLUSIONS***

After preliminary work to explore feasibility with the Assurance Maladie teams, the number of eligible doctors was estimated at 2,400 GPs who saw more than 100 different patients in 2018. The average number of DDDs dispensed per doctor in 2018 was 7,671, with a standard deviation of 5,360. In 2018, the average number of DDDs dispensed per doctor was 7671, with a standard deviation of 5360. Due to cluster randomisation, an inflation factor must be applied to calculate the number of subjects. In a previous study in the Loire Atlantique and Vendée regions [47], the average number of GPs per practice was 1.9. We set an intra-class correlation coefficient of 0.05 [50].

With an alpha risk of 5% and power of 80%, we can then demonstrate a significant difference if we have at least an average difference of 450 DDD per doctor (over the 12 months following the DAM visit) between the arms being compared.

- 1. ***Expected degree of statistical significance***

The alpha risk is set at 5%.

- 1. ***Method for taking into account missing, unused or invalid data***

All analyses will be performed on an intention-to-treat (ITT) basis.

The volumes of systemic antibiotics dispensed attributed to doctors who ceased their activity (end of career, moving outside the region, death, etc.) during the study follow-up period will be replaced by multiple imputation. A sensitivity analysis of the per-protocol population will also be carried out, excluding these doctors, as well as those who did not receive a visit from the DAM, and those who were not computerised.

For doctors who have not been visited by the DAMs, the start date of the data collection period will be allocated by drawing lots, and staggered over the 3 months (start date based on the visit dates available for the 3 randomisation arms).

- 1. ***Randomisation***

The Assurance Maladie will generate a list of GP practices that meet the inclusion criteria. It will then randomise the GP practices on a 1:1:1 basis. Doctors will be assigned to the randomisation arm to which their practice has been allocated.

1. **SAFETY / UNDESIRABLE EFFECT**

In the regulatory sense and within the meaning of the Jardé Law, this study does not constitute research involving human subjects. In the regulatory sense and within the meaning of the Jardé Law, this protocol does not involve any change in the usual management of patients, so any adverse events or effects observed will be unrelated to the study.

1. **MEDICO-ECONOMIC STUDY**

This section presents the objectives and methodology of the efficiency analysis, based on a cost-effectiveness analysis. The economic evaluation methodology follows the 2011 recommendations of the French National Authority for Health [49].

- 1. ***Objective, perspective and time horizon of the cost-effectiveness analysis***

The study involves a cost-effectiveness analysis comparing the delivery of antibiotics over a 12-month period in current practice, compared with two alternative strategies

i) an intervention carried out by the Délégués d'Assurance Maladie (Health Insurance Representatives) in general practices, combining a regular visit (with awareness-raising on antibiotic resistance, work on preconceived ideas, feedback on practices) and a demonstration of the use of Antibioclic,

1. an intervention carried out in the same way by the Délégués d'Assurance Maladie, but without Antibioclic being demonstrated.

This analysis will be carried out from the point of view of the Assurance Maladie Obligatoire over a 12-month period.

Costs will be estimated on the basis of information retrieved from the SNDS databases extracted by the Assurance Maladie in Pays de la Loire. The resources consumed will be valued at Assurance Maladie prices for drugs.

Given that two strategies will be compared with current practice, it may be necessary to estimate several incremental cost-effectiveness ratios (ICERs). A dominance analysis will be carried out to check whether certain interventions are dominated, i.e. more costly and less effective than another intervention. The average incremental cost-effectiveness ratio will be presented, together with its 95% confidence interval.

Univariate and multivariate sensitivity analyses will be carried out to check the robustness of the results to changes in assumptions about certain study parameters.

- 1. ***HEALTHCARE CONSUMPTION AND VALUATION***

The costs of the three strategies compared will be estimated in three stages [48]:

. collection of resources consumed,

. the unit monetary value of resources,

. estimated total costs.

Deliveries and costs of systemic antibiotics will be estimated using information retrieved from SNDS databases extracted by the Assurance Maladie in Pays de la Loire. The resources consumed will be valued at Assurance Maladie prices for drugs.

The resources used as part of the study will include :

. the costs associated with preparing and holding the training course,

. training time for DAMs,

. the development of communication material,

- the time it takes to create a digital visual aid,

- time for role-playing,

- the time it takes to write a sales pitch,

- time to rewrite the memo cards.

. time doctors

. the average duration of use of Antibioclic by doctors

We will take into account antibiotic dispensing based on the doctor's prescription and the monitoring of activity over a 12-month period. The costs will be calculated using Assurance Maladie tariffs.

This will be done by combining the results of the two previous stages. Given the 12-month time horizon, the costs will not be discounted. We will present the average costs per patient in each arm.

- 1. ***Efficiency data***

Effectiveness will be measured by the ratio between deliveries of defined daily doses (DDD) observed in the Assurance Maladie databases and the WHO defined daily doses between the different scenarios in the analysis [46-47].

We will look at defined daily doses (DDD) of all types of systemic antibiotics for all patients per 100 consultations [51]. To do this, we will stratify by specific antibiotic types (ie, tetracyclines, quinolones, β. lactamine/penicillin, other β. lactamine, other antibacterials, unspecified, etc.).

Finally, we will stratify the patient populations into 4 classes: young children (0 to 6 years), children over 6 years and adolescents (7 to 17 years), young adults (18 to 65 years) and finally the elderly (over 65 years) and between men and women.

- 1. ***Presentation of results***

In accordance with ISPOR recommendations on economic evaluation [52,53], we will present the results of the medico-economic analysis as follows:

. average antibiotic costs per arm and the difference in costs with its 95% confidence interval

. Mean efficacy per arm and the difference in efficacy with its 95% confidence interval

As costs are not generally distributed according to a normal distribution, the incremental cost-effectiveness ratio and its 95% confidence interval will be estimated using the non-parametric bootstrap technique.

Finally, sensitivity analyses will be carried out to study the robustness of the results on certain key parameters of the study.

1. **ADMINISTRATIVE AND REGULATORY ASPECTS**
   1. ***Right of access to source data and documents***

The data extracted as part of the study will not be nominative, whether it concerns physician data or patient data. They will only be transmitted to the sponsor or any person duly authorised by the sponsor, and, where applicable, to the authorised health authorities, under conditions which guarantee their confidentiality (see 7.1.COLLECTION AND PROCESSING OF STUDY DATA).

There is no provision for access to source documents. This means that there will be no export of nominative data outside the usual health insurance databases.

The data collected during the trial will be processed electronically, in compliance with CNIL requirements.

- 1. ***Computerised data and submission to the CNIL***

Data processing will be recorded in the Nantes University Hospital's RGPD register.

The data collected during the study will be kept in a computer file that complies with the amended French Data Protection Act of 6 January 1978, Law No. 2018-493 of 20 June 2018 on the protection of personal data and Regulation (EU) 2016/679 of the European Parliament and of the Council of 27 April 2016 on the protection of individuals with regard to the processing of personal data and on the free movement of such data (RGPD).

The protocol falls within the scope of the MR004 Reference Methodology to which Nantes University Hospital complies.

- 1. ***Inspection / Audit***

This study may be subject to inspection or audit. The sponsor and/or the investigators must be able to give the inspectors or auditors access to the data.

- 1. ***Ethical considerations***
     1. **Procedures for obtaining consent**

**This is a study outside the scope of the Jardé Law** [54,55]. Research involving the human person does not include *research aimed at evaluating the methods used by healthcare professionals or teaching practices in the healthcare field.*

*In addition, the study provides for an exemption from the requirement to inform patients by reference to international standards as defined by the IRB in the light of the following criteria:*

*"(1) the research is designed to evaluate possible changes in public health programs, and (2) the research can not practicably be carried out without the waiver". [56]*

- - 1. **Ethics Committee**

The experimental protocol will be analysed in accordance with regulatory ethical imperatives. This is a study outside the scope of the Jardé Law. The project will be subject to the prior opinion of an Ethics Committee that rules on non-RIPH projects, such as that of the Collège National des Généralistes Enseignants (CNGE).

- 1. ***Amendments to the protocol***

Substantial changes must be set out in an updated dated version of the protocol.

- 1. ***Publication rules***

Authors will be determined on the basis of their contribution to the study. The coordinating investigator will draw up the list of authors.

Any publication resulting from this research will carry the mention: "This study was supported by a grant from the French Ministry of Health (PREPS 2019)".

1. **PROJECT COORDINATION ARRANGEMENTS**

The project was co-constructed with the Pays de la Loire health insurance scheme.

- 1. ***Role of each team***
- **Department of General Medicine, University of Nantes**

. Coordinates the study

. Responsible for the scientific development of the study: review of the international scientific literature, design, methodology, etc.

. Co-responsiblefor developing the intervention

. Co-responsible for organising training meetings and presentations to health insurance delegates

. Responsible for statistical data analysis

. Responsible for the scientific promotion plan

- **Department of Infectious and Tropical Diseases, Nantes University Hospital**

. Participates in the coordination of the study

. Providing scientific expertise in infectiology and antibiotic resistance

. Participate in the scientific development of the study: international scientific literature, design, methodology, etc.

. Co-responsible for developing the intervention

. Co-responsible for organising training meetings and presentations to Health Insurance delegates

. Participate in the interpretation of results and in the scientific development plan

- **Health insurance**

. Co-responsible for coordinating the study

. Responsible for the inclusion of participating GPs

. Co-responsible for organising training meetings and presentations to the Health Insurance Representatives

. Responsible for coordinating the DAM network

. Responsible for randomisation

. Responsible for extracting and collecting data from the SNDS.

- **MEDQUAL**

. Expertise in infectiology and antibiotic resistance

. Expertise on training needs in infectiology and antibiotic resistance.

- **ANTIBIOCLIC**

. Expertise in infectiology and antibiotic resistance

. Design of the Antibioclic tool (made available to GPs as part of the intervention)

. Expertise on the needs and practices of general practitioners .

- 1. ***Steering Committee***

All the teams take part in the steering committee. This committee defines the general organisation and progress of the project, coordinates information and decides what to do in unforeseen circumstances.

*References*

1. Cassini A, Högberg LD, Plachouras D, et al. Burden of AMR Collaborative Group. Attributable deaths and disability-adjusted life-years caused by infections with antibiotic-resistant bacteria in the EU and the European Economic Area in 2015: a population-level modelling analysis. Lancet Infect Dis. 2019;19:56-66. doi: 10.1016/S1473-3099(18)30605-4.
2. de Kraker ME, Stewardson AJ, Harbarth S. Will 10 million people die a year due to antimicrobial resistance by 2050? PLoS Med. 2016;13:e1002184.
3. Davies SC, Fowler T, Watson J, et al. [Annual Report of the Chief Medical Officer: infection and the rise of antimicrobial resistance.](https://www.ncbi.nlm.nih.gov/pubmed/23489756/) Lancet. 2013;381(9878):1606-9.
4. Centers for Disease Control and Prevention (CDC). Vital signs: carbapenem-resistant Enterobacteriaceae. MMWR Morb Mortal Wkly Rep 2013;62:165-70.
5. Bell BG, Schellevis F, Stobberingh E, et al. A systematic review and meta-analysis of the effects of antibiotic consumption on antibiotic resistance. BMC Infect Dis. 2014;14:13.
6. Gelband H, Pant S, Gandra S, et al. The state of the world's antibiotics, 2015. Washington D.C: Center for Disease Dynamics, Economics Policy. 2015. <http://www.cddep.org/publications/state_worlds_antibiotics_2015#sthash.2fHwn4BD.dpbs>
7. Centers for Disease Control and Prevention. Antibiotic resistance threats in the United States, 2013. Atlanta, Georgia: Centres for Disease Control and Prevention, US Department of Health and Human Services, 2013.
8. Costelloe C, Metcalfe C, Lovering A, et al. Effect of antibiotic prescribing in primary care on antimicrobial resistance in individual patients: systematic review and meta-analysis. BMJ. 2010;340:c2096. doi: 10.1136/bmj.c2096
9. ANSM. Antibiotic consumption in France in 2016
10. Maugat S, Berger-Carbonne A. Antibiotic consumption and resistance in France. Joint report by ANSM, Assurance Maladie, Santé Publique France. November 2018. Available on

<Https://www.santepubliquefrance.fr/content/download/145982/2132831>

1. Steinman MA, Landefeld CS, Gonzales R. Predictors of broad-Spectrum antibiotic prescribing for acute respiratory tract infections in adult primary care. JAMA 2003;289:719-25.
2. Cadieux G, Tamblyn R, Dauphinee D, et al. Predictors of inappropriate antibiotic prescribing among primary care physicians. CMAJ 2007;177.
3. Dooling KL, Shapiro DJ, Van Beneden C, et al. Overprescribing and inappropriate antibiotic selection for children with pharyngitis in the United States, 1997-2010. JAMA Pediatr. 2014;168:1073-4. doi: 10.1001/jamapediatrics.2014.1582.
4. McDonagh MS, Peterson K, Winthrop K, et al. Interventions to reduce inappropriate prescribing of antibiotics for acute respiratory tract infections: summary and update of a systematic review. J Int Med Res. 2018;46:3337-57. doi: 10.1177/0300060518782519.
5. Butler CC, Dunstan F, Heginbothom M, et al. Containing antibiotic resistance: decreased antibiotic-resistant coliform urinary tract infections with reduction in antibiotic prescribing by general practices. Br J Gen Pract. 2007;57:785-92.
6. National Institute for Health and Clinical Excellence. Respiratory tract infections - antibiotic prescribing. Prescribing of antibiotics for self-limiting respiratory tract infections in adults and children in primary care. July 2008. https://www.nice.org.uk/guidance/cg69/evidence/full-guideline-196853293.
7. Ivers N, Jamtvedt G, Flottorp S, et al. Audit and feedback: effects on professional practice and healthcare outcomes. Cochrane Database Syst Rev. 2012;6:CD000259.
8. Hoa NQ, Thi Lan P, Phuc HD, et al. Antibiotic prescribing and dispensing for acute respiratory infections in children: effectiveness of a multi-faceted intervention for health-care providers in Vietnam. Glob Health Action. 2017;10:1327638. doi:10.1080/16549716.2017.1327638.
9. van der Velden AW, Pijpers EJ, Kuyvenhoven MM, et al. Effectiveness of physician-targeted interventions to improve antibiotic use for respiratory tract infections. Br J Gen Pract. 2012;62:e801-7. doi: 10.3399/bjgp12X659268.
10. Gulliford MC, Prevost AT, Charlton J, et al. Effectiveness and safety of electronically delivered prescribing feedback and decision support on antibiotic use for respiratory illness in primary care: REDUCE cluster randomised trial. BMJ. 2019;364:l236. doi:10.1136/bmj.l236
11. Sharp AL, Hu YR, Shen E, et al. Improving antibiotic stewardship: a stepped-wedge cluster randomized trial. Am J Manag Care. 2017;23:e360-e365.
12. McDonagh MS, Peterson K, Winthrop K, et al. Interventions to reduce inappropriate prescribing of antibiotics for acute respiratory tract infections: summary and update of a systematic review. J Int Med Res. 2018;46:3337-57. doi: 10.1177/0300060518782519.
13. Goossens H, Ferech M, Vander Stichele R, Elseviers M, Group EP; ESAC Project Group. Outpatient antibiotic use in Europe and association with resistance: a cross-national database study. Lancet. 2005;365(9459):579-587.
14. Drekonja DM, Filice GA, Greer N, et al. Antimicrobial stewardship in outpatient settings: a systematic review. Infect Control Hosp Epidemiol. 2015;36(2):142-152.
15. Attali C et al. Presumed viral respiratory infections. How to prescribe fewer antibiotics? Rev Prat Med Gen 2003;17:155-60.
16. Le Corvoisier P, Renard V, Roudot-Thoraval F, et al. Long-term effects of an educational seminar on antibiotic prescribing by GPs: a randomised controlled trial. Br J Gen Pract. 2013;63:e455-64. doi: 10.3399/bjgp13X669176.
17. Ferrat E, Le Breton J, Guéry E, et al. Effects 4.5 years after an interactive GP educational seminar on antibiotic therapy for respiratory tract infections: a randomized controlled trial. Fam Pract. 2016;33:192-9. doi: 10.1093/fampra/cmv107.
18. Meeker D, Linder JA, Fox CR, et al. Effect of Behavioral Interventions on inappropriate antibiotic prescribing among primary care practices: a randomized clinical trial. JAMA 2016;315:562-70. doi: 10.1001/jama.2016.0275.
19. MacDougall C, Polk RE. Antimicrobial stewardship programs in health care systems. Clin Microbiol Rev 2005;18:638-56.
20. Hemkens LG, Saccilotto R, Reyes SL, et al. Personalized prescription feedback using routinely collected data to reduce antibiotic use in primary care. A Randomized Clinical Trial. JAMA Intern Med. 2017;177:176-83. doi:10.1001/jamainternmed.2016.8040
21. Horwood J, Cabral C, Hay AD, et al. Primary care clinician antibiotic prescribing decisions in consultations for children with RTIs: a qualitative interview study. Br J Gen Pract. 2016;66:e207-13. doi:10.3399/bjgp16X683821.
22. Razon Y, Ashkenazi S, Cohen A, et al. Effect of educational intervention on antibiotic prescription practices for upper respiratory infections in children: a multicentre study. J Antimicrob Chemoth. 2005;56:937-40.
23. Christakis DA, Zimmerman FJ, Wright JA, et al. A randomized controlled trial of point-of-care evidence to improve the antibiotic prescribing practices for otitis media in children. Pediatrics. 2001;107:e15.
24. Margolis CZ, Warshawsky SS, Goldman L, et al. Computerized algorithms and pediatricians' management of common problems in a community clinic. Acad Med. 1992;67:282-84.
25. <https://antibioclic.com>
26. Jeanmougin P, Aubert JP, Le Bel J, et al [Antibioclic: a tool for rational antibiotic therapy in primary care]. Rev Prat 2012,62:978.
27. Jeanmougin P, Delory T, Mara C, et al. ANTIBIOCLIC: what is the profile of users? (P-74). In: *RICAI*. Paris, France; 2014.
28. Heslot C, Guery MP, Imbert P, Epaulard O, Wintenberger C. Duration of antibiotic therapy prescribed by general practitioners for acute community-acquired pneumonia. P-228. In: RICAI. Paris, France; 2017.
29. De Faucal S, Ouazzani K, Ferrand H, et al. Improving antibiotic therapy practices in primary care with Antibioclic. P-229. In: *RICAI*. Paris, France; 2017.
30. Sharp AL, Hu YR, Shen E, et al. Improving antibiotic stewardship: a stepped-wedge cluster randomized trial. Am J Manag Care. 2017;23:e360-5.
31. Litvin CB, Ornstein SM, Wessell AM, et al. Use of an electronic health record clinical decision support tool to improve antibiotic prescribing for acute respiratory infections: the ABX-TRIP study. J Gen Intern Med. 2013;28:810-6. doi: 10.1007/s11606-012-2267-2.
32. <https://assurance-maladie.ameli.fr/carrieres/metiers/efficacite-systeme-sante/delegue-assurance-maladie>
33. <https://assurance-maladie.ameli.fr/carrieres/metiers/efficacite-systeme-sante/delegue-assurance-maladie>
34. WHO Collaborating Centre for Drug Statistics. ATC/DDD Index 2019. Dec 2018. <https://www.whocc.no/atc_ddd_index/>
35. WHO. DDD: Definition and general considerations. https://www.whocc.no/ddd/definition_and_general_considera/. Accessed July 30, 2019
36. French National Institute for Statistics and Economic Studies. Study area. <https://www.insee.fr/fr/information/2114631> (accessed 13 aug 2019)
37. Rat C, Pogu C, Le Donné D, et al. Effect of physician notification regarding nonadherence to colorectal cancer screening on patient participation in fecal immunochemical test cancer screening: a randomized clinical trial. JAMA 2017;318:816-24.
38. SPILF - Recommendations for the management of community-acquired urinary tract infections in adults - 2017 update. http://www.infectiologie.com/fr/recommandations.html
39. Haute Autorité de Santé, economic evaluation and public choice department. Methodological choices for the economic evaluation of the HAS. HAS ; 2011 oct.
40. <http://id.erudit.org/iderudit/007859ar>
41. Drummond MF, Sculpher MJ, Claxton K, et al. Methods for the Economic Evaluation of Health Care Programmes. 4th ed. Oxford: Oxford: Oxford University Press, 2015.
42. Hemkens LG, Saccilotto R, Reyes SL, et al. Personalized prescription feedback to reduce antibiotic overuse in primary care: rationale and design of a nationwide pragmatic randomized trial. BMC Infect Dis. 2016;16:421. doi:10.1186/s12879-016-1739-0.
43. Husereau D, Drummond M, Petrou S, et al; CHEERS Task Force. Consolidated Health Economic Evaluation Reporting Standards (CHEERS) statement. BMJ. 2013;346:f1049. doi: 10.1136/bmj.f1049.
44. Decree No. 2017-884 of 9 May 2017 amending certain regulatory provisions relating to research involving the human person. Available at: https:[//www.legifrance.gouv.fr/eli/decret/2017/5/9/AFSP1706303D/jo/texte](https://www.legifrance.gouv.fr/eli/decret/2017/5/9/AFSP1706303D/jo/texte)
45. Jouannin A, Mamzer MF, De Fallois M, et al. Validation of a research classification tool for general medical interns based on the "Jardé" law. Exercer. In Press.
46. Code of Federal Regulations. TITLE 45. Public welfare. Department of health and human services. Part 46. Protection of human subjects, available at https://www.hhs.gov/ohrp/regulations-and-policy/regulations/45-cfr-46/index.html#46.116
