# Supplementary material for: Effect of a Feedback Visit and a Clinical Decision Support System Based on Antibiotic Prescription Audit in Primary Care: Multiarm Cluster-Randomized Controlled Trial
Source: J Med Internet Res. 2024 Dec 18;26:e60535. doi: 10.2196/60535 (PMC11694052; doi:10.2196/60535)
Supplement: Multimedia Appendix 6 [file jmir_v26i1e60535_app6.pdf]

Comité Ethique du CNGE
155 rue de Charonne 75011 PARIS
Courriel : comite-ethique@cnge.Fr
Tél : 01 75 62 22 90

N°IRB / IRB00010804

A Paris, le 18 Juillet 2019,

Objet : Avis du Comité Ethique du CNGE concernant l'étude "Effet sur les prescriptions d'antibiotiques d'une intervention multifacette menée par les délégués de l'assurance maladie dans les cabinets de médecine générale"

AVIS 110719107

Les auteurs souhaitent réaliser une étude sur les effets sur la prescription d'antibiotiques d'une intervention multifacette menée par les délégués de l'assurance maladie dans les cabinets de médecine générale, comportant la démonstration de l'utilisation d'un outil internet d'aide à la décision : Antibioclic.
Le comité d'éthique a donné un avis favorable à la réalisation de ce projet.

Cédric RAT
Pour le Comité Ethique du CNGE
