# Supplementary material for: Effect of a Feedback Visit and a Clinical Decision Support System Based on Antibiotic Prescription Audit in Primary Care: Multiarm Cluster-Randomized Controlled Trial
Source: J Med Internet Res. 2024 Dec 18;26:e60535. doi: 10.2196/60535 (PMC11694052; doi:10.2196/60535)
Supplement: Multimedia Appendix 7 [file jmir_v26i1e60535_app7.docx]

Multimedia Appendix 7:

Total volume of systemic antibiotics dispensed in defined daily doses (DDD) per participating GP after a 12-month follow-up (Per protocol analysis).

| **Arm** | **Number of General Practitioners**  *Per-Protocol population** | **Total volume of systemic antibiotics dispensed**  Mean *(Standard Deviation)* | **Absolute difference (95% CI)** | ***P*** |
| --- | --- | --- | --- | --- |
| CDSS based visit group | 596 | 4877 (*3322.0*) | -209.9 [-320.6 ; -99.5] | < 0.001 |
| Standard visit group | 571 | 4725 (*3549.2*) | -127.4 [-230.7 ; -24.0] | 0.016 |
| Control | 733 | 4930 *(3467.1)* | Reference | Reference |

** GPs who ceased their activity during the follow-up period or GPs in the CDSS based visit group or Standard visit group who did not receive a visit from a HIR were deleted from the Per Protocol population.*
